# Quality of FDM 3D Printed Medicines for Pediatrics: Considerations for Formulation Development, Filament Extrusion, Printing Process and Printer Design

**DOI:** 10.1007/s43441-021-00354-0

**Published:** 2021-11-26

**Authors:** Julian Quodbach, Malte Bogdahn, Jörg Breitkreutz, Rebecca Chamberlain, Karin Eggenreich, Alessandro Giuseppe Elia, Nadine Gottschalk, Gesine Gunkel-Grabole, Lena Hoffmann, Dnyaneshwar Kapote, Thomas Kipping, Stefan Klinken, Fabian Loose, Tristan Marquetant, Hellen Windolf, Simon Geißler, Tilmann Spitz

**Affiliations:** 1 Institute of Pharmaceutics and Biopharmaceutics, Heinrich Heine University Düsseldorf, Universitätsstr. 1, 40225 Düsseldorf, Germany; 2 Merck Healthcare KGaA, Frankfurter Str. 250, Darmstadt, Germany; 3 Merck Life Science KGaA, Frankfurter Str. 250, Darmstadt, Germany; 4 Gen-Plus GmbH & Co. KG, Staffelseestr. 6, München, Germany; 5 Laboratory for Manufacturing Systems, University of Applied Sciences Cologne, Betzdorfer Str. 2, 50679 Cologne, Germany

## Abstract

3d printing is capable of providing dose individualization for pediatric medicines and translating the precision medicine approach into practical application. In pediatrics, dose individualization and preparation of small dosage forms is a requirement for successful therapy, which is frequently not possible due to the lack of suitable dosage forms. For precision medicine, individual characteristics of patients are considered for the selection of the best possible API in the most suitable dose with the most effective release profile to improve therapeutic outcome. 3d printing is inherently suitable for manufacturing of individualized medicines with varying dosages, sizes, release profiles and drug combinations in small batch sizes, which cannot be manufactured with traditional technologies. However, understanding of critical quality attributes and process parameters still needs to be significantly improved for this new technology. To ensure health and safety of patients, cleaning and process validation needs to be established. Additionally, adequate analytical methods for the in-process control of intermediates, regarding their printability as well as control of the final 3d printed tablets considering any risk of this new technology will be required. The PolyPrint consortium is actively working on developing novel polymers for fused deposition modeling (FDM) 3d printing, filament formulation and manufacturing development as well as optimization of the printing process, and the design of a GMP-capable FDM 3d printer. In this manuscript, the consortium shares its views on quality aspects and measures for 3d printing from drug-loaded filaments, including formulation development, the printing process, and the printed dosage forms. Additionally, engineering approaches for quality assurance during the printing process and for the final dosage form will be presented together with considerations for a GMP-capable printer design.

**Supplementary Information:**

The online version contains supplementary material available at 10.1007/s43441-021-00354-0.

## Introduction

The general principle of pharmaceutical 3d printing, or additive manufacturing, renders this approach a promising candidate for the automated manufacturing of solid pediatric medicines [1]. Solid medicines have significant benefits over the use of liquids for the treatment of children. They provide a high microbial stability, good chemical and physical stability, enable controlled release properties and demonstrate high dosing accuracy [2]. With common manufacturing approaches, dosages can be varied only incrementally in certain ranges. 3d printing enables manufacturing of medicines with precise and fully variable dosing. Dosage forms are printed layer-by-layer in a shape predefined in a computer aided design (CAD) software. In theory, every imaginable size and shape can be printed. A direct consequence of this approach is the ability to modify the dosage simply and conveniently, a lack of which is widely recognized as a major hurdle in pediatric therapy [3]. Besides the inherently possible size adaption, which is key to improve acceptability [4], 3d printing techniques also allow the manufacturing of small batches down to a single individual dosage form for a given patient.

While several 3d printing techniques exist and are investigated for pharmaceutical use [5], *fused deposition modeling* (FDM) emerges as one of the most interesting technologies for the manufacturing of pediatric medicines. In FDM, filaments, drug-loaded polymer wires, are fed into the printhead of the 3d printer. In the printhead, the filament is heated and extruded through a nozzle on a temperature-controlled print bed. A kinematic system allows movement of the printhead in *x*-, *y*-, and *z*-direction respective to the printhead, enabling the layer-wise deposition of the polymer-drug matrix until the dosage form is printed. Filaments are manufactured in a hot-melt extrusion (HME) step, which has to be performed industrially due to the required equipment, environment, and process understanding. This results in two main advantages. Firstly, a properly developed formulation and hot-melt extrusion process result in a high-quality intermediate that undergoes proper quality testing. Secondly, the active pharmaceutical ingredient (API) is bound in a polymer matrix within the filament, significantly reducing the risk of drug exposure for the professional operating the printer. The combination of these advantages makes FDM the most promising candidate for manufacturing of (pediatric) medicines also in decentralized settings, e.g., hospitals, compounding centers and community pharmacies. Other technologies require either the manufacturing of aqueous intermediates that cannot be prepared easily industrially due to the risk of contamination during storage [6] or the handling of powders in the final printing step, e.g., binder jetting and selective laser sintering [5], which bears a high risk of operator exposure. If a semi-solid formulation is prepared in decentralize settings, proper quality control of the API distribution within the semi-solid and printed dosage forms requires significant analytical effort that cannot be performed for individual batches.

While many publications cover proof-of-concept studies of novel dosage form designs and approaches to formulation and process development [7, 8] quality consideration of excipients, formulations, processes, filaments and medicines are frequently mentioned but rarely formalized. This lack was recognized by the United States Pharmacopeia (USP) and the International Association for Pharmaceutical Technology (APV) who co-organized a 4-day workshop on quality and standards considerations of 3d printed medicines (Homepage workshop). Even though quality aspects are mentioned for dosage forms for adult drug therapy, no publications about the quality of pediatric 3d printing are available until now.

This publication aims to remedy this issue. The authors are members of the PolyPrint project [9] a project funded by the German Federal Ministry of Education and Research (BMBF). The project consortium exists of the companies Merck KGaA and Gen-Plus, the Laboratory for Manufacturing Systems of the University of Applied Sciences Cologne and the Institute of Pharmaceutics and Biopharmaceutics at Heinrich Heine University Düsseldorf. In the project, novel polymers for pharmaceutical FDM 3d printing are developed and thoroughly tested and benchmarked. Additionally, a novel type of FDM printer is developed to enable high-quality and cGMP compliant 3d printing of medicines. Here, we reflect on the status of the complete manufacturing process of 3d printed pediatric medicines beginning with the raw materials and ending with the final dosage form. We highlight existing shortcomings and provide guidance based on the experience gathered in the PolyPrint project.

## Key Attributes of Raw Materials

### Quality Aspects of Pharmaceutical Excipients

Pediatric formulation developments are obliged to follow the guidance of the EMA ensuring the overarching goal: “The development of pediatric formulations and presentations is necessary to ensure that children of all ages and their caregivers have access to safe and accurate dosage forms of medicines.”[10]

More detailed information is provided in the “Guideline on pharmaceutical development of medicines for pediatric use” [11]. In general, solid oral dosage forms such as tablets and capsules can offer advantages of greater stability, accuracy of dosing and improved portability over liquid formulations. To assure suitable swallowability the size of tablets and capsules should be kept as small as possible [2].

The choice of excipients plays an important role in pediatric formulation development, both for safety and acceptability of the resulting dosage forms. The physiology of neonates and infants differs considerably from that of adults. They exhibit significantly different clearance and volume of distribution as well as differences in the metabolic profile [12]. Prominent excipient examples for the resulting challenges and issues are propylene glycol or sorbitol in infants. Also, polyethylene glycol (PEG)—a useful additive for filament plasticity and solubility enhancement—needs careful consideration regarding maximum intake. While studies confirm safe use of, e.g., PEG 3350–4000 as laxative, undesired laxative effects and potential gastrointestinal disorders limit the use of PEG to 10 mg/kg/d [13].

Looking at FDM based 3d printed formulations, usually the polymer makes up more than 50% of the formulation. Given the comparatively high intake of these excipients, the safety of polymers and additives (glidants, plasticizers) in pediatric formulations is a very important factor, especially if (partial) degradation of the polymer in the gastrointestinal tract (GIT) is to be expected. Therefore, not only polymer but also degradants and synthesis constituents of the polymer need to be carefully integrated into the safety assessment for pediatric medications. To date, several pharmaceutical polymers, such as methacrylates and ethylcellulose, are commercialized in pediatric products. Unfortunately, most polymers are used in comparatively low amounts as coating agents for taste masking and release modification [14]. Little information is available for polymers used as matrix agent and related high daily intake. Although observed adverse reactions from coated formulations might be used to prevent further incompatibilities, the maximum acceptable intake for children is critical and not easily derived from toxicological studies performed in adults. An important tool for assessing the safety of relevant excipients is the STEP database (Safety and Toxicity of Excipients for Pediatrics) [15].

In addition to safety, the taste sensation of excipients needs to be carefully considered. Polymers and additives should be taste- and odorless and ideally offer opportunities for obscuration of taste (see subsection on taste masking).

The important decision factors affecting the use of excipients are summarized by Yochana et al.: “Excipients for pediatric formulations should be carefully selected with reference to the age of the pediatric population, ADME developmental changes, and duration of treatment to ensure safety and efficacy of such formulations in pediatric population.” [16]

### Polymer Requirements—Limitations in FDM

**Figure 1:**
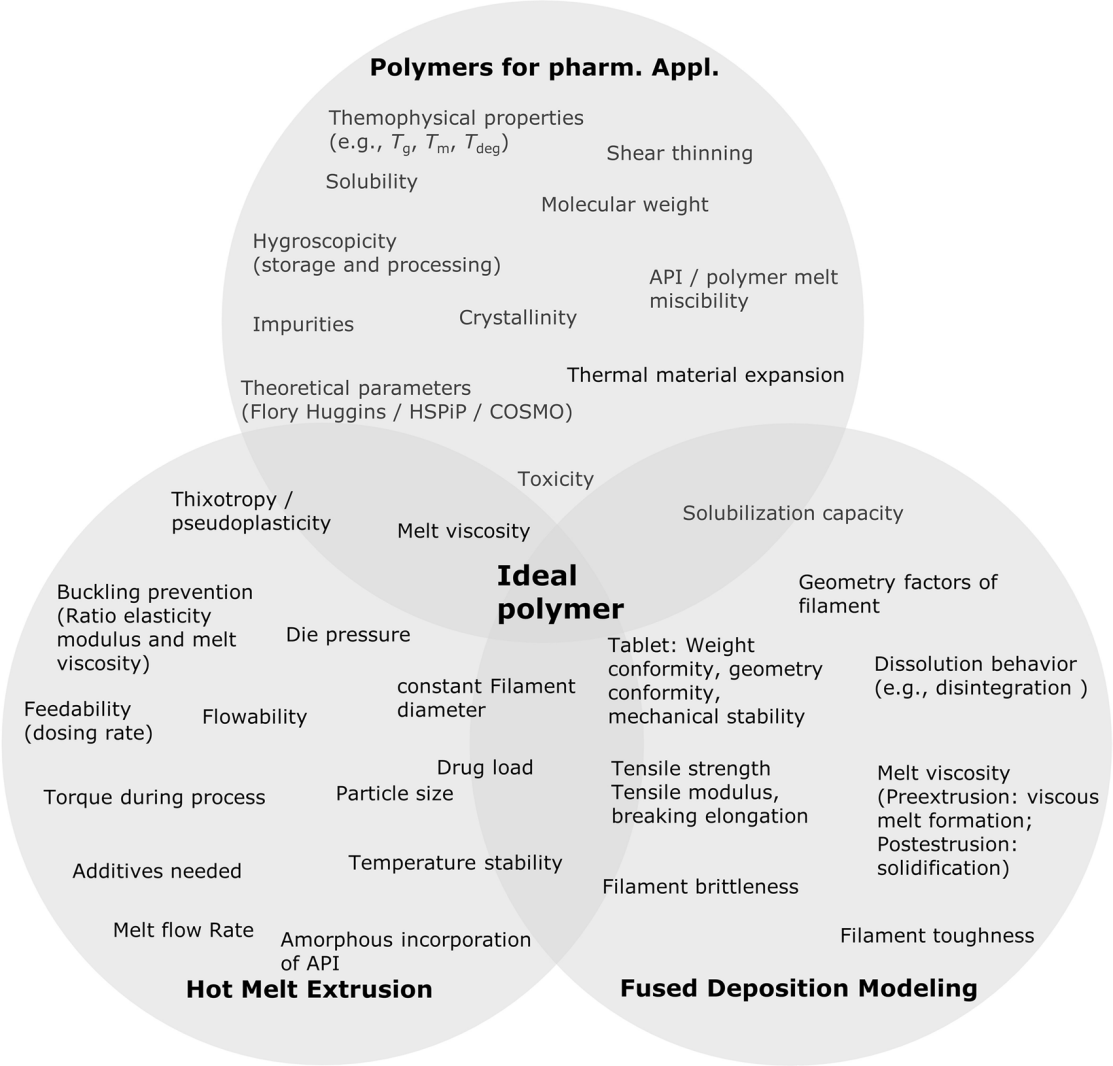
Selection of Parameters That are Relevant for Pharmaceutical Application of Polymers, Particularly in HME and FDM.

In recent years, the application of thermoplastic polymers in pharmaceutical development of 3d printed products via FDM has gained increasing interest. A multitude of different material requirements need to be fulfilled by the polymer for these applications, as illustrated in Fig. 1 (further details on these parameters can be found in the supplementary information in Table S1). Here, we summarized relevant properties and parameters, which influence the suitability of given polymers or APIs, respectively. For an overview of different polymer families and a selection of marketed products in the field of hot-melt extrusion, the reader is referred to Simoes et al. [17, 18]. 3d printing using the FDM technique requires further polymer prerequisites [19] in addition to the parameters important in HME development, which typically depend on the product properties and the utilized API. During an FDM 3d printing process, the polymeric filament is subject to significant mechanical forces. A specific mechanical profile is required due to “pinching” of these filaments between two feeding gears in the printhead. Filaments carrying a high Young’s modulus (> 300 N/mm^2^, depending on printhead) can be conveyed without breakage or deformation [20, 21]. At the same time, the tensile strength and the brittleness of the extrudates are crucial parameters for successful printing [19, 21–28]. The latter of which may be assessed using the three-point bending test (breaking distance > 1–1.5 mm [21, 22] and breaking stress > 2941–3126 g/mm^2^ [22]). Nasereddin et al. evaluated a selection of the most commonly used polymers in FDM and developed a screening method to assess their brittleness including these parameters and thus evaluate printability [24].

### Taste Masking

Taste is an important sensation to be considered in pharmaceutical development. Taste aversiveness might impact patients’ compliance and medication adherence. Sensory components of both the olfactory and the gustatory sensations have to be distinguished. Whereas substances which should develop the smell as an olfactory signal need to be volatile under the conditions of drug administration, the gustatory system is directly based on the tongue comprising different types of taste buds and papillae where the sensory receptors and ion channels for salty, sour, sweet, bitter, and umami taste are located. Depending on the properties of the poorly tasting components, various taste-masking techniques are available [14]. In pharmaceutical printing technologies, most of the proposed taste-masking approaches can be applied although scientific papers or patents are scarce:

- One obvious approach is to mask the unpleasant taste of a printed object by the addition of differently tasting excipients, e.g., sweet carbohydrates (sucrose, fructose, glucose), sugar alcohols (mannitol, xylitol, sorbitol) or artificial sweeteners (saccharine, aspartame, cyclamate or acesulfame). An olfactory signal can be added to the printing formulation using volatile substances such as menthol or more complex organic or synthetic flavors [29]. However, it should be noted that one or more components of these mixtures will partly evaporate during the manufacturing and over storage time changing taste sensation as a key property to be controlled in stability studies.
- Unpleasant tasting ingredients can be physically bound within inclusion complexes, e.g., by use of cyclodextrins, or to polyelectrolytes (anionic or cationic polymers) which can also be part of the solid matrix. Entrapping by the printed polymers may be sufficient for taste masking of bitter compounds [30, 31].
- High viscosity hydrophilic polymers may prevent fast hydration and dissolution of the dosage form, thereby reducing the migration to the taste receptors on the tongue and the resulting taste sensation.
- Barriers inside or outside the printed dosage form may shield against quick dissolution and saliva contact.

The taste-masking effect of the applied pharmaceutical measures are usually demonstrated by using advanced dissolution setups [30] or electronic tongues in vitro [32], and human taste panels [31] or animal experiments like the BATA (brief-access taste aversion) model in vivo [33].

## Hot-Melt Extrusion of Intermediates for Pediatric 3d Printing

### Filament Extrusion—a Question of Homogeneity

The filament required for FDM 3d printing is generated as endless strand via twin-screw hot-melt extrusion. Filament extrusion comprises multiple individual unit operations and processes that must be considered to obtain an overview of relevant quality attributes. To meet quality attributes of products and establish robust processes, the Food and Drug Administration (FDA) recommends quality-by-design (QbD) approaches for formulation and process development [34]. This led to different implementations of QbD in pharmaceutical melt extrusion processes [34–36]. As mentioned in the section on polymer requirements, the mechanical properties of filaments must allow proper feeding and extrusion from the printhead. Additionally, diameter homogeneity and API distribution are much more important compared to regular hot-melt extrusion processes. Usually, the obtained extrudate is milled or pelletized and a subsequent homogenization of the individual particles is performed. In FDM, the filament is commonly kept intact and printed as it exited the extrusion die. This means that diameter and API distribution inhomogeneities directly reflect in the printed amount of filament and API. This can be an issue for regularly sized dosage forms [37] and even more so for pediatric medicines of lower dose and mass. In this case, even small variations of diameter and API distribution can lead to non-compliance with monographs on the uniformity of dosage units and must be avoided. The API distribution is influenced primarily by the powder feeding process and hot-melt extrusion, the diameter homogeneity by the hot-melt extrusion process.

### Powder Feeding

**Figure 2:**
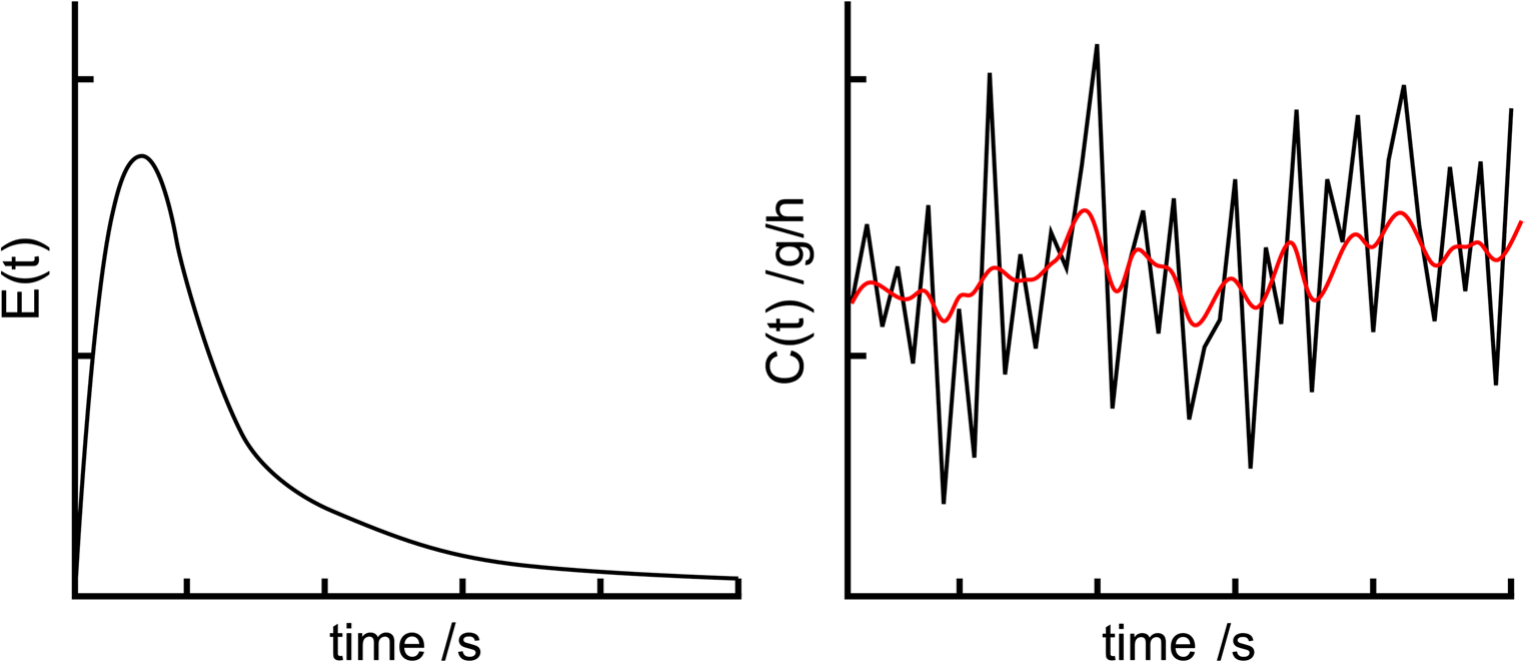
Exemplary Drawing of: (left) a Typical Residence Time Distribution Function of a Hot-Melt Extrusion Process; (right) Fluctuations of the Feed Rate (Black) and Output Fluctuations After Extruder (Red).

In twin-screw filament extrusion processes feeding of polymers, solid additives and APIs is a critical aspect. Unlike single-screw extruders, twin-screw extruders are run partially filled. Thus, the material flow inside the extruder depends on the flow rate of the feeder used. Process parameters like the specific feed load (SFL) and residence time distribution (RTD) are directly influenced by the feeding [38]. In Fig. 2 left, a typical residence time distribution curve of a filament extrusion process is shown. On the right, feeding fluctuations are shown in black and the resulting output fluctuations after extrusion are shown in red. The reduction in fluctuations demonstrates the mixing and homogenizing abilities of extruders. As the reduction is not absolute, feeding should be as homogeneous as possible, to reduce output variations.

Several types of dispensing devices are available for feeding bulk solids. Vibrating trays or screws are a widespread method of conveying the material [39]. Simple devices feed in volumetric mode at a constant actuating variable. In contrast, loss-in-weight or gravimetric feeders are equipped with an integrated load cell that detects fluctuations in the feed rate. The actuating variable is adjusted via a control mechanism, leading to a compensation of fluctuations [40]. Material properties as well as the target feed rate must be considered in selection of the most suitable dosing device [41]. Low dosing rates and poor flow properties result in particularly high demands on equipment attributes [42]. Matarazzo et al. has provided a checklist to assist in the selection of proper feeder equipment [43].

Bulk solid feed is evaluated in several studies usually by using an external scale where the fed material is collected. Data analysis of the dosing curve or its integral can be conducted using statistic parameters like measure of dispersion or target-actual-ratio of moving measures of central tendency [44, 45]. Another way is using discrete Fourier transform of dosing fluctuations, which provides information about the materials dosed [46].

### Extruding Filaments as Intermediates

The efficiency of the melting process of polymers in HME depends on the properties of the excipients and the extruder design. In general, polymers with low melt viscosities and high thermal conductivities exhibit a more efficient melting process. Changes in the screw design are often necessary to improve the melting process of the powders and to improve mass flow of the melt through the extruder. Otherwise, solid material may block the screws transiently, which can result in increased torque if the melting process is incomplete.

**Figure 3:**
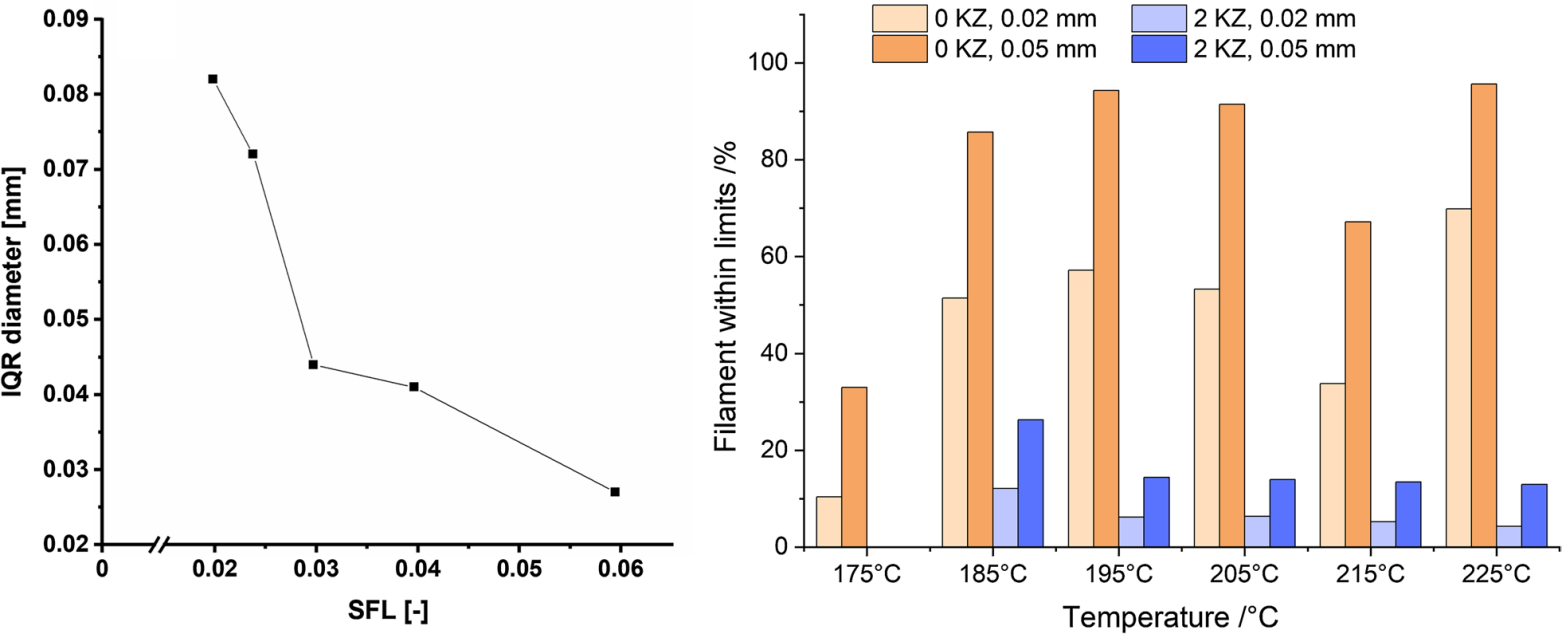
(Left) Interquartile Ranges of Diameter Measurements During Extrusion Correlated with SFL of the Extrusion Process [37], (Right) Amount of Filament Within ± 0.02 and 0.05 mm Specification With or Without Two Kneading Zones (KZ 1: 4 × 90°,4 × 60°, 2 × 30°, KZ 2: 8 × 60°, Unpublished Data).

Ponsar et al. highlighted the effect of the extruder barrel fill level on filament homogeneity. The higher the fill level, the lower are the fluctuation of the mean diameter (Fig. 3 left) [37]. Frequently, as diameter fluctuations are not necessarily normal distributed, the inter quartile range of the diameter is used to describe fluctuations. Besides having a measure for fluctuations, it is as important to set limitations for said variations. Usually, deviations of ± 0.02 mm or 0.05 mm from the set value are considered tolerable. By varying the temperature, the polymers, the process setting and the screw configuration in an extruder, the limits are met with varying degrees of success. In Fig. 3 right, the same formulation was extruded at different temperatures and with different screw designs (no kneading zones or two kneading zones). The best batch was extruded at a high temperature of 225 °C with no kneading zone and the worst at the same temperature with two kneading zones. These data show the decrease of filament diameter within the ± 0.02 mm or 0.05 mm specification when adding two kneading zones. This observation indicates the importance of a continuous melt flow in the extruder, which is better provided by a screw configuration of only conveying elements. When adding kneading elements, the melt is retained before the kneading zones until enough pressure is build up by the following melt. To increase the homogeneity of the filament diameter further, a melt pump can be attached between the end of the extruder barrel and the die. The purpose of this attachment is to stabilize inevitable melt fluctuations that occur within the hot-melt extrusion process. The pump aligns those fluctuations by metering the melt flow to a very constant rate and therefore a very constant pressure level [47]. This leads to an increase of filament homogeneity since the fluctuations mentioned before are reduced drastically. This was shown by in-process monitoring of filament diameters [48].

After extrusion, it must be considered how to properly cool down the obtained filament. Commercial FDM filaments such as acrylonitrile butadiene styrene copolymer (ABS) or polyether ether ketone (PEEK) are not water soluble and can therefore be cooled down in a water bath. Polymers for pharmaceutical FDM applications are frequently at least partially water soluble and they contain one or more APIs. Consequently, cooling in a water bath cannot be performed, even though it is a highly effective and efficient cooling process. For pharmaceutical applications, proper cooling can be achieved by either passive cooling on a conveyor belt at atmospheric conditions or by using an air ring or air tunnel [49].

While polymer melt is being pushed out of the nozzle, a phenomenon can occur known in HME as “die swell”. Die swell is the expansion of molten polymer to a larger diameter than determined by the die itself, resulting in a filament thicker than desired. This effect is mainly related to the energy preserved by compression and force alignment of polymer chains being forced through the die, followed by the relaxation of those chains when exiting the die again [50]. The viscoelastic behavior of the melt as well as process parameters are major factors when die swell shall be reduced [37, 51]. A reduction of die swell can be achieved by increasing the temperature at the die. Even with thorough optimization, a larger mean diameter than desired will frequently result. To further adjust the mean diameter after extrusion, a pulling unit, e.g., a conveyor belt or the haul-off unit of a winder [37] can be implemented. The speed of haul-off units is variable and defines the final mean diameter of the filament, which can be wound or used as individual strands. Commercial filament diameters are typically 1.75 or 2.85 mm. For pharmaceutical purposes, a lower diameter is beneficial, as potential inhomogeneities of diameter and content will have less of an impact relatively.

**Figure 4:**
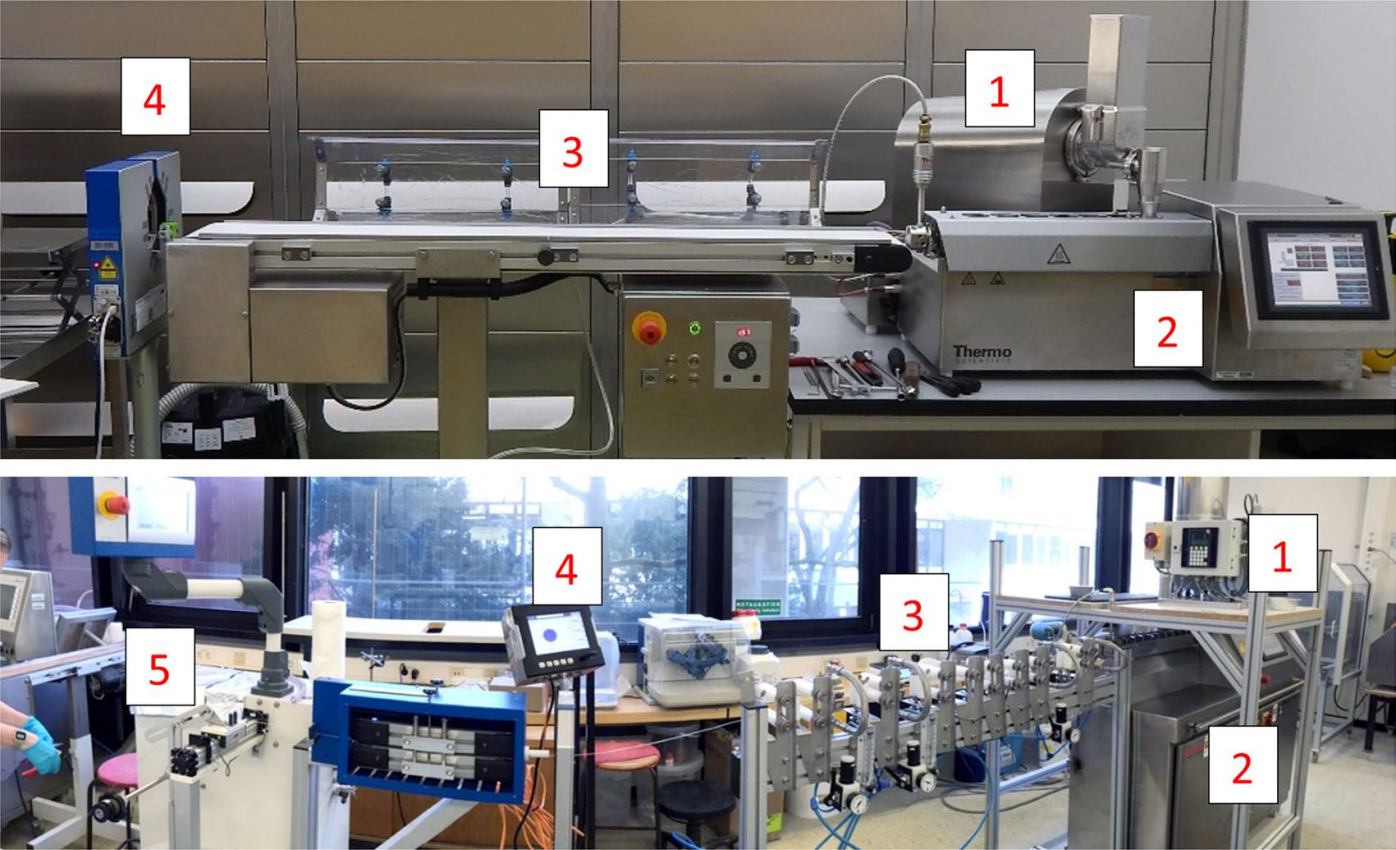
Two Filament Production Lines. (1) Gravimetric Feeders, (2) Twin-Screw Extruders, (3) Cooling via Conveyor Belt (Top) and Ring Air-Knives (Bottom), (4) Three-Axis Laser Micrometers, (5) Winding Unit.

In Fig. 4, two prototype extrusion lines are shown. They consist of gravimetric powder feeders, twin-screw hot-melt extruders, cooling units (conveyor belt or cooling line with ring air-knives), laser-based diameter measurement system and optionally a filament winder.

### Characterization of Filaments

To evaluate, optimize and monitor the process of filament production, different analytical tools can be used off-line and in-line.

### Off-line Characterization

A simple and useful approach is the visual assessment of API-loaded filaments. This way, it is frequently possible to initially assess potential degradation via color changes and possible recrystallization of the active ingredient(s) especially for higher drug loadings and APIs that exhibit thermal sensitivity. As already discussed in the section on polymer requirements, the mechanical properties of filaments are an important factor for the feedability of the formulation that must be analyzed. The mechanical properties of filaments may change over time due to enthalpy relaxation [21] or because the included excipients are hygroscopic. Water absorbed during processing or storage is a powerful plasticizer that lowers the glass transition temperature. Is does not only affect the mechanical properties and appearance, but also drug stability, may induce degradation, and needs to be quantified for this reason [52, 53]. In vitro dissolution as per compendial monographs is used to determine the amount of drug dissolved over time and thereby to assess the performance of the formulation (filament/tablet) in regard to release behavior [54]. For the content determination and examination of homogeneous drug distribution as well as characterization of related substances within filaments and tablets, most frequently HPLC analysis is used [55].

### In-line Characterization of Filaments via PAT

The physicochemical properties of filaments produced by HME are crucial for the 3d printing process. Quality and performance of the 3d printed tablet and can be examined with PAT tools like spectroscopy, rheometry and optical coherence tomography (OCT) [56]. These tools enable capturing real-time information of process and filament properties during HME non-destructively. Some of the data can be easy to interpret, e.g., diameter and sphericity of filaments determined via multi-axes laser scanning modules (see Fig. 4). Some can be difficult to interpret and may require the preparation of multivariate, quantitative models, for example spectral information. Independent of the data complexity, it can be utilized to monitor the process and initiate corrective actions to reach a desired state and potentially to allow real-time release [57]. In the following, relevant technologies are listed.

### In-line Spectroscopy

UV–vis spectroscopy has been used and established as PAT tool in HME. Spoerk et al. used in-line UV–vis spectroscopy as an analytical tool for characterizing of active ingredients (Estradiol, Estriol, Ibuprofen) and polymer matrices (ethylene vinyl acetate, Eudragit RL-PO). The studies focused on the quantification of the drug for cleaning-in-place strategies [58]. Wesholowski et al. have investigated in-line UV–vis spectroscopy as a PAT tool for preparing solid dispersions of two APIs (carbamazepine and theophylline) with one polymer (copovidone) [59]. The obtained results revealed the suitability of the implemented tool to quantify the drug load in a typical range for pharmaceutical applications. The range of linearity differed with different formulation and was 5–30% for carbamazepine, whereas that for the theophylline formulations was 2.5–10%. They reported that the efforts to evaluate data was minimal due to univariate data analysis and in combination with a measurement frequency of 1 Hz, the system is sufficient for the real-time data acquisition. In-line near infrared (NIR) spectroscopy has also been used to investigate drug–polymer interactions and to validate a method for continuous API quantification during HME processing [60]. Vo et al. demonstrated the use of Fourier transform NIR spectroscopy in conjunction with multivariate analysis (MVA) for in-line API concentration monitoring during a HME process [61]. In this study, they used ketoprofen as model drug, Eudragit L100-55 as matrix polymer and stearic acid as processing aid. A principal component analysis (PCA) model was used to monitor the process state shift in response to disturbances of parameters, such as temperature and material feed rate. Thus, an NIR based quality monitoring methodology can be easily transferred from process development to manufacturing. Saerens et al. evaluated the suitability of Raman spectroscopy as PAT tool for the in-line determination of API concentration and the polymer-drug solid state during HME process [60]. They used different concentration of metoprolol tartrate (10%, 20%, 30%, and 40%) with Eudragit RL-PO mixtures, which were extruded and monitored in-line in the die using Raman spectroscopy. A PLS model was developed and validated, which allowed the real-time API concentration determination. They also investigated application of Raman spectroscopy in solid-state characterization and found that the mixtures containing solid solution showed broadened Raman peak compared with the solid dispersion.

### In-line Rheometry

In-line measurements of the rheological characteristics play an important role in real-time monitoring of torque, influence of drug load, and effect of formulation ingredients on the process. The real-time evaluation of rheology data in the extrusion process can be determined by pressure drop inside an extruder die connected to suitable instruments. In-line rheological characterization can enhance process control and understanding [62].

### Optical Coherence Tomography (OCT)

OCT, a non-invasive method, is used as an off-line tool for semi-transparent and turbid media. It can be applied to measure parameters such as surface properties of filaments and layer thicknesses, e.g., of coating layers or filaments produced in hot-melt co-extrusion, and uniformity [63]. Koutsamanis and Eggenreich et al. reported the application of OCT to evaluate the integrity of the core/membrane interface and membrane thickness of vacuum compression molding formulations containing progesterone with ethylene vinyl acetate polymer [64].

### Characterization of the Solid State

Even though some of the above-mentioned analytical tools can determine certain aspects of solid-state properties, other approaches are commonly used that provide a better understanding of materials. The solid state of an API incorporated in a polymer matrix can have a large impact on the performance of the final dosage form in terms of dissolution rate and bioavailability [65]. Poorly soluble APIs, which make up a large proportion of potential drug candidates [66], can be formulated as amorphous solid dispersions (ASD) where the crystalline structure of the API is broken up and the resulting molecular dispersions are stabilized by a polymer matrix. In contrast, an API can also be incorporated in filaments maintaining a crystalline structure [67, 68]. The presence or absence of crystalline structures strongly influences printability, such as mechanical [69] and rheological properties of filaments [70]. Consequently, the assessment of crystallinity in filaments is important in process development, quality control and stability studies. Even though this assessment can be supported by in-line measurements, traditional techniques are more widespread.

The formation and stability of the ASD is influenced by solubility and miscibility of the API and the polymer matrix [71]. Thermal and mechanical energy uptake during manufacturing facilitates the dispersion and reduces the number and size of crystal nuclei, which may lead to premature precipitation of API in vivo or reduce physical stability during storage. To maintain the solid state during shipment and storage is important for the ASD itself, but for FDM the second heating cycle during printing needs to be considered, additionally. The thermal impact may not only impair the chemical stability of the formulation but can also lead to recrystallisation of API [28].

Several techniques can be applied to determine the solid state. Differential scanning calorimetry (DSC) as well as well as X-ray powder diffraction (XRPD) are well-established analytical methods to investigate the solid state of API dispersed in polymer matrices [72]. One caveat is the limit of detection of crystalline fractions in mostly amorphous systems [73]. The detection of small traces of crystalline fraction is possible by the use of polarized light microscopy [74]. However, this method lacks quantitative determination and selectivity.

In regard to the assessment of crystallinity in intermediate and final product the manufacturing process should be considered end to end for FDM printed solid dosage forms.

## FDM Printing at Site of Care—Stricter Requirements for Dosing Precision and Quality Control

3d printing based on FDM has been state of the art for years and is used primarily in the consumer sector but also in industrial environments. Particularly in industry, a quality demand is placed on the products to be printed from the ground up. Unlike in pharmaceutical industry, however, the focus is primarily on geometric aspects.

Different consumer 3d printers are already being used in pharmaceutical research. One of several issues with off-the-shelf printers is that the amount of active ingredient processed cannot be verified. Thus, the quality of the pharmaceutical products is not verifiable. In contrast to classical manufacturing methods, 3d manufacturing is slow and only few dosage forms are printed [5]. Therefore, destructive quality control approaches are not profitable and in-line testing is unavoidable. Furthermore, there is hardly any system on the market that meets the cleaning requirements of pharmaceutical equipment [75].

In addition to the common requirements of mechanical engineering for the development and market placement of production machines, special requirements are part of the GMP guidelines [76]. For these reasons, it is imperative to rethink 3d printer design and adapt it to the needs of pharmaceutical manufacturing. The following sections highlight some of the most critical components.

### Motion System and Overall Printer Design

The most common design in FDM 3d printing is the Cartesian printer, but other forms like the delta printer and the polar printer exist [77]. Cartesian printers operate by linear movement of the printhead in x-, y-, and z-direction respective to the print bed. In most cases, the axes, motors, and drives are designed for general industrial and mechanical engineering purposes and the requirements of the pharmaceutical industry are not considered. For example, many of the moving parts, which are usually lubricated, are not encapsulated and are, therefore, exposed to potential contaminants from filament and product. Since it is required for pharmaceutical production that all surfaces in contact with the product are cleanable, these elements do not meet the GMP standard [78].

**Figure 5:**
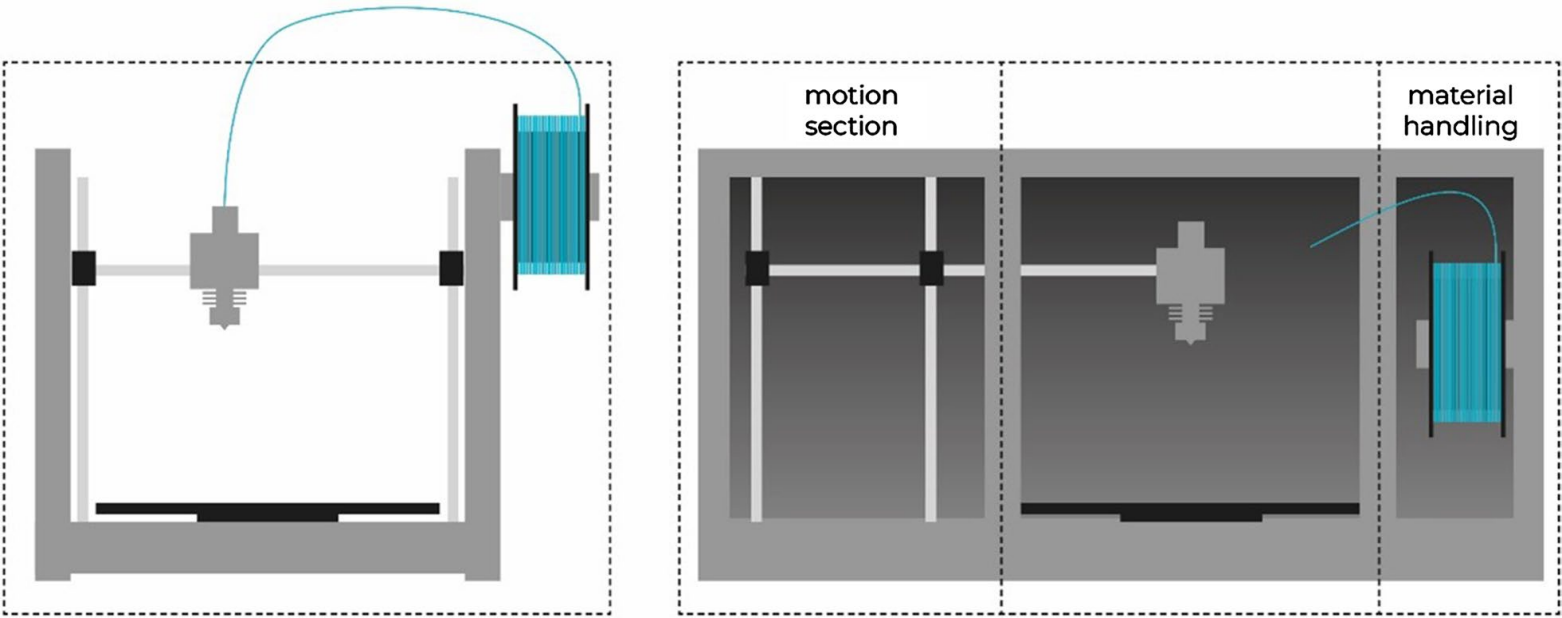
Schematic of an Off-the-Shelf 3D Printer (Left) and a 3D Printer with Separate Build, Motion, and Material Handling Section (Right).

During the development of new machines all requirements for the system need to be defined beforehand. In addition to the basic functions for a 3d printer almost all machines are designed to be as compact and as inexpensive as possible. To achieve this, many functions are implemented in a small space. When looking at existing printing systems under the prerequisites of the GMP guideline, several problems become apparent. In regular 3d printing systems, all subsystems such as material handling, material processing, build plate, and motions system are implemented openly in a very confined space. For a GMP-compliant implementation, however, it is recommended to separate all elements and to design individual and well controlled areas (Fig. 5).

For industrial and non-pharmaceutical applications, the print chamber usually does not have to be kept particle-free or sanitized. Axis systems for moving print head or print bed can be placed directly in the printing space. Since outgassing, particle detachment and other sources for (cross-)contamination must be contained or avoided during the print job, this arrangement is not applicable for GMP printers. The printing chamber should be as isolated as possible from all moving elements. In addition, surfaces should not have complex geometries or sharp angles to ensure cleanability.

In addition to the risk of contamination of the printer parts, attention must also be paid to the safety of the operator. During the processing of APIs, the user may be exposed to harmful chemicals. For example, in the case of outgassing, it must be ensured that substances cannot endanger the user. For this purpose, the printer should be equipped with appropriate protective devices such as air filters. In the pharmaceutical sector, little research has been done on the possible safety aspects of using 3d printing for the manufacture of pharmaceutical products [78].

Here, it is advisable to use approaches from industrial 3d printing as a starting point. Powder-based printing technologies in particular place great emphasis on user safety. The GMP guidelines stipulate that all surfaces in contact with the product must be made of approved and cleaning-resistant materials, and that these must not have any edges, joints, undercuts or similar [79]. For this reason, all elements should be milled or machined from a single piece of material, if possible.

### Feeding Mechanism, Filament, and Filament Storage

In commercially available printers, filament is conveyed in the extruder by two counter-rotating rollers. To increase the conveying force, at least one of the rollers is a gear wheel. This approach to filament transport is not suitable for pharmaceutical materials. The force exerted on the filament might become too high, resulting in slippage. Slippage, in turn, leads to small, usually statically charged filament grains that accumulate in the cavities of the feed roller and on other elements in the printhead. At high conveying resistances, i.e., high melt viscosities, this effect can even lead to breakage and creation of dislodged filament pieces. The consequence of this behavior is that the extruder must be cleaned extensively to avoid cross-contamination. In particular, the complex geometry of the gear wheel(s) with its many cavities prevents efficient cleaning. In addition, damage to the material leads to an undefined geometry of the filament and, thus, to an uncontrolled quantity of deposited material. Breakage of the filament will lead to printing process failure and manual intervention will be required to restart the process.

Traditional FDM 3d printing is based on a spool-based filament supply system. Technical polymers for classical FDM printing are designed and manufactured to display sufficient flexibility to be wound on a spool, but also enough stiffness to be processed by a standard feeding mechanism. As described in the section on key attributes of excipients, pharmaceutical polymers often do not allow reliable feeding and printing easily due to their brittleness or undesirable deformation behavior. A filament provision and supply system must be developed that can handle a greater diversity of mechanical properties. To achieve this, both the bearing and the extruder technology must be completely revised.

Up to 450 m of filament can be wound onto a spool. When printing multiple large components this is an advantage. For the production of small dosage forms in lower quantities this is not necessary. If a lower amount of material is required, a smaller filament supply that is used up quicker reduces potential issues with the storage stability. Particularly in view of the API cost, smaller units of filaments are to be preferred. In addition, cross-contamination of filament must be avoided during handling so that encapsulation of the filament is necessary. For this reason, the currently selected filament geometry (“endless”) and the bearing units (coils) must be questioned.

The material storage, commonly designed as filament reel, should also be redesigned as part of a separate area. This is realized by some commercial printers that have cartridge systems, but large amounts of material are still wound on spools. We recommended to reduce the amount of material stored in or on the material accumulator. With reduced material amounts, coiled-up, long filaments strands that require feeding rollers or gears are not necessary, solving multiple issues with the current printer design. Omitting spools enables a new design of the storage system which can offer hermetic encapsulation of the filament. This would allow filament storage and transport under controlled conditions, similar to a tablet in a blister. Initial approaches can already be found in printers from the company Stratasys [80]. Yet, these are not suitable for pharmaceutical manufacturing machines without significant modifications.

If filaments are not a continuous long strand anymore and new bearing units are designed, the conveying mechanism needs to be revised, too. Roller or gear-based feeding mechanisms should not be used for this purpose as they facilitate cross-contamination. Piston based mechanisms similar to those already used in certain bioprinters [81, 82] would be a superior approach, as slippage and breakage could be prevented.

### Hotend and Coldend

One of the most central parts in a 3d printer is the hotend. With the help of electrical heating, the polymer is melted and extruded through a nozzle. Conventional hotends are optimized for high throughput and printing speeds. Technical polymers allow processing at temperatures well above the melting or glass transition temperature to reduce duration of melt formation. The result is a high temperature gradient from the core to the edge of the filament [83]. The use of additive manufacturing in the pharmaceutical environment, on the other hand, requires processing that is particularly gentle on the material, as many APIs are thermo-labile. Nevertheless, high printing speeds must still be achieved for a productive process. It is necessary to optimize the hotend in terms of uniform heat input to reduce the heat strain put on pharmaceutical filaments.

The other components of a common printhead are also not suitable for use in the pharmaceutical manufacturing. To compensate for the high temperatures, the upper parts of the printing core, the coldend, are cooled to prevent or reduce softening of the filament before the actual hotend. Commonly, active air cooling with cooling fins is used. Due to their complex and fine geometry these parts are particularly difficult to clean and increase the risk of cross-contamination. As the hotend is located directly above the product, evaporation of residual solvents, plasticisers and other volatile components is to be expected. They will be distributed in the printing chamber via the cooling air, further increasing the risk of cross-contamination and reducing cleanability. Pharmaceutical print heads must be completely cleanable. Purging with cleaning filaments, what is the common procedure in research, will not suffice to prevent cross-contamination. Since the material is fed through coldend and hotend, all elements that come into contact with the product must be cleaned without residues after each use and before each material change. To avoid changing the complete print head, a system design similar to the design of hot-melt extruders is recommended. Similar to the barrels, the printhead should be demountable and the material touching parts easily accessible [58]. The coldend of the printhead is placed in the print chamber as well and its cooling fins cannot be cleaned properly. Switching to water cooling would solve this issue and provide a more accurate control of the temperature. This has been realized in some non-pharmaceutical systems such as the x500pro from German RepRap [84].

### Sensors and Quality Control

A few years ago, 3d printing gained a detrimental reputation of being usable only for prototyping, due to frequent print failures, limited resolution, anisotropic mechanical properties, low production speeds and rough surface finish [85]. The reason for this is a lack of process and quality control. Even though the implementation of in-line quality is beginning in some 3d printing technologies, in FDM such methods are still at the experimental or research stage. The focus is mostly on thermal monitoring (melt pool analysis in selective laser sintering / melting) and layer monitoring [86]. Even though some of these approaches can also be used in a pharmaceutical context, they focus mostly on quality attributes for other manufacturing industries. Relevant pharmaceutical quality attributes cannot be captured with such systems. The use of the sensors applied for control issues only allows accurate control and regulation of the process. However, a quality statement regarding the solid state, API content or printed quantity is not possible. For 3d printers to be used for pharmaceutical manufacturing in the future, additional measures must be undertaken in addition to adapting the mechanical components. A major point is the quality control of the printed product.

Various types of defects in 3d printed parts are described in the literature [86, 87]. While structural integrity is key for technical applications of FDM, the doses of incorporated API in the final dosage form is the crucial parameter for medication manufactured by FDM. Especially medications to be used by children need to be manufactured in an accurate way, because the doses for children are typically lower and small deviations in the content of a dosage form result in higher relative over or underdosing potentially harming the patient.

To improve the quality of dosage forms, process control has to be improved as well. In general, three groups of parameters can be identified for in-process control.

1. Machine parameters derived from the control electronics. For example, motor and heater current, temperature of nozzle and cooling zone, vibrations etc.
2. Monitoring of the extruded volume or mass of the filament, either derived from measurements described in 1 or measured by dedicated sensors attached to the printer.
3. Non-destructive chemical analysis of the raw material and / or intermediate and final printed product.

The monitoring of machine parameters can be implemented in industrial control systems and do not necessarily rely on additional sensors which might lead to higher machine costs in the end. It has been described in literature to use the current of the feeding motor to detect a blocked nozzle [87, 88]. Chemical degradation and under-extrusion related to blocked nozzles is a major concern for the quality and accurate dosing of pharmaceutical dosage forms. In another example, Becker et al. [87, 89] used accelerometers to monitor the state of the printer and detect fluctuations in the flow of extruded raw material during the printing process. These substitute parameters can only be used if comparative data are available of the printing process for a specific raw material on a specific printer limiting the application to well understood processes. To circumvent these issues, dedicated sensors can be introduced into the printing system to directly measure the extruded volume or mass. Calculation of the printed dosage may act as a valuable in-process control, assuming that the active ingredient is homogenously distributed in the raw material.

Optical systems were described to measure the distance between printed object and nozzle detecting under-extrusion [90, 91] providing error detection during a print process. It is also possible to monitor the movement and quality of filament by a camera [92]. Dosage forms with defects can then be discarded after the printing process and documented in a batch report for documentation. Of course, specialized sensors as well as integrated balances measuring the actual printed mass of filament could be an option to further improve the accuracy of 3d printed oral dosage forms, assuring the quality. For printing at the site of care, implementation of feedback loops based of the obtained data to automatically adjust the printing process of each individual dosage form will enable to meet the claimed dose and lead to an efficient manufacturing process with reduced waste and higher yields resulting in fast supply of high-quality medication to the patients.

Focusing more on the final product than the process itself, chemical analysis of the API incorporated in the printed dosage form could enable real-time release in a clinical printing setup. Different spectroscopic methods were described in the literature to analyze pharmaceutical dosage forms without destroying the samples. It was shown that NIR-chemical imaging of 3d printed objects can be used to measure the amount of printed API [93]. Transmission Raman measurements were reported in literature to investigate the amorphous and crystalline fraction of fenofibrate in solid oral dosage forms [94]. Such methods could be used in future pharmaceutical 3d printers to assure the quality of amorphous solid dispersions. Chemical analysis of printed objects can lead to full batch real-time release of medications printed at site of care ensuring that the quality of the final product was not negatively influenced by the printing process. Downsides of non-destructive chemical imaging are high costs and large equipment, which might not be easily integrated in the printing platform.

Still, FDM as a digitally controlled manufacturing process opens the opportunity to integrate multiple sensors to not only monitor the quality of the printing process and product but furthermore adjusting critical process parameter on the fly to resemble a true rapid manufacturing process.

## 3d Printed Dosage Forms for Pediatric Use

### General Consideration

Only few solid dosage forms have been investigated for their acceptability in children [95]. Of those, even less can be manufactured by 3d printing: minitablets, orodispersible films, (orodispersible) tablets, and capsules. A definite advantage of 3d printing is a freedom of design previously not possible. This is demonstrated by many novel dosage form designs [7, 96]. It is likely that such novel designs will also demonstrate high acceptability in children, e.g., because of more appealing coloring and dimensions. However, this has not been demonstrated in clinical trials and this section will focus mostly on the above-mentioned dosage forms. As the resolution of FDM printing is limited, small capsules suitable for pediatric treatment are not sensible to manufacturing via this route. Similarly, printing of orodispersible tablets and minitablets has not been established, yet.

### Minitablets and Small Oblong Tablets

Tablets of < 4 mm diameter are usually considered to be minitablets [97]. As they have demonstrated acceptability in neonates, infants and children [2, 98] minitablets of 2 mm diameter are developed and manufactured more frequently than larger ones. Accurate printing of small objects is challenging in general. A typical nozzle diameter is 0.4 mm and tablets with a diameter of 2 mm are only five times larger than the nozzle diameter. These small geometries are on the lower limit of what is possible with the FDM process [99] and dimensional accuracy is difficult to achieve. Due to the small surface area of a single layer the cool down period of the material before the nozzle passes an area a second time is short, which may lead to insufficient mechanical stability of already printed layer. Several strategies are suitable to circumvent such issues. Reduction of print speed is generally associated with higher dimensional accuracy and improved surface quality. However, throughput and productivity will decrease with lower print speed. The manufacturing order of objects on one build plate can also be changed from sequential printing (complete all layers of one object, then moving on to the next object) to layer-wise printing where the printing layer is changed after the specific layer of all objects is completed. While cooling time per object layer is associated with beneficial effects on dimensional accuracy, frequent changes between objects may introduce additional classes of printing errors like stringing and blobs [100]. Every additional travel movement comes with the risk of oozing filament and, therefore, inaccuracy of dose. The dosing of smaller tablets is even more challenging than with larger objects because the relative change of incorporated API due to printing defects is more significant. Krause et al. printed objects with a diameter of 4 mm, 3 mm, 2 mm and 1.5 mm with decreasing tablet mass and calculated the acceptance value according to Ph. Eur. 2.9.40. While the standard deviation of tablet mass was higher for the largest objects, their acceptance value was lower compared to smaller tablets. These results show that dosing accuracy is especially important for mini tablets and low dose drug forms [99].

Ayyoubi et al. printed spherical tablets with a diameter of 6 mm with channels to improve dissolution rate [101]. Small oblong tablets (9 mm × 5 mm × 4 mm (width × length × height)) were manufactured for children > 6 years old by Fanous et al. [102].

Another aspect besides the dimensionally accuracy is the geometric flexibility. Geometrical flexibility offers the opportunity to increase the compliance in pediatric patient as well as to reduce the resistance of taking medication in children. The reason lies in the possibility of 3d printing for personalized medicine to choose the color, shape and design of the tablet according to the child's wishes [103]. Scoutaris et al. imitated chewable STARMIX® sweets by printing objects in the shape of a heart, ring, bottle, bear and lion, which contained the model substance indomethacin, hypromellose acetate succinate (HPMCAS) and polyethylene glycol (PEG) as polymers. The aim for the development of this pediatric dosage forms with the STARMIX® design via hot-melt extrusion (HME) and FDM 3d printing was also to enhance the palatability [104].

Besides the flexibility in geometry FDM can also be used to manufacture layer-wise polypills [105]. Multiple APIs were printed into one solid oral dosage form. In case of the layer-based FDM process, chemical compatibility of these APIs is not as limiting as in traditional manufacturing processes since the compounds are separately printed into different compartments of the dosage form. The flexibility of a computer-controlled manufacturing process opens the possibilities to match the exact needs for pediatric patients, but deep understanding of the underlaying processes and optimized print settings are necessary to ensure high quality of the final product.

### Orodispersible Films

Orodispersible films are accepted by infants and children [95, 106, 107] and are dosage forms of choice for patient centric applications. The European Pharmacopoeia defines orodispersible films as solid oromucosal preparations intended for the administration in the mouth, where they disperse rapidly to deliver active substances (Ph. Eur. Monograph “Oromucosal preparations”). Dose adaption is possible by (1) modifying the API concentration in the formulation, (2) adapting the film thickness, and (3) by cutting films to the desired size, as both thickness and size defines the amount of incorporated API. However, the cutting approach can be accompanied by material waste and is prone to human errors.

Manufacturing routes of orodispersible films include solvent casting [108–110] and 2d and 3d printing technologies. In 2D printing [111, 112], the printing fluid consists of the drug dissolved in a suitable solvent or dispersed in a dispersant, which is printed onto a substrate which contains polymer(s) and additives (e.g., plasticizers, flavors) and is made in a separate manufacturing step. As for the solvent casting technique, the process parameters (drying temperature, humidity) need to be precisely controlled, as they significantly influence the final film properties [113, 114].

3d printing offers a waste-less route of precise manufacturing medicines for children. Jamróz and colleagues accurately printed orodispersible films containing aripiprazole [115], whereas Ethezazi et al. printed multi-layered films containing individual layers with paracetamol, ibuprofen and a taste-masking agent [29]. Cho et al. applied a variation of FDM printing to prepare an orodispersible film containing the poorly water soluble drug olanzapine [116]. They heated a polymer-API mixture until it melted and used pneumatic extrusion to drive the printing process, a approach similar to the one published by Musazzi et al. [108]. In another study, a bi-layer film was FDM printed with a mucoadhesive chitosan layer and drug containing layer and an ethyl cellulose backing layer that formed a permeation barrier, thus creating a unidirectional drug release [117].

Even though none of these studies directly investigated the suitability of FDM 3d printing to individualize the dose, they demonstrated sufficient mechanical properties to enable robust handling and acceptable accuracy that strongly hints at technological proficiency to produce pediatric orodispersible films. However, acceptability of orodispersible films was assessed with solvent-casted films and the different appearance of FDM printed films will have to be investigated separately in future studies.

### Dosage Form Characterization

To ensure that printed dosage forms meet the requirements, physical properties need to be characterized, and the homogeneous distribution of the API has to be controlled to guarantee that patients receive the necessary therapeutical amount of API. For physical characterization, various tests are listed in the pharmacopeia: test for friability (Ph. Eur. 2.9.7), crushing strength (Ph. Eur. 2.9.8) and disintegration (Ph. Eur. 2.9.1). To check the homogeneity of the drug distribution, the content of the API in the tablets is determined via the uniformity of the mass or content (Ph. Eur. 2.9.5 / 2.9.6) or the uniformity of dosage units (Ph. Eur. 2.9.40). In addition, it is tested how the drug is released from the tablet over time (Ph. Eur. 2.9.3).

However, FDM printed tablets have different physical properties than compressed tablets, so further methods have been developed for physical characterization. Often, the printed tablets are less porous than the pressed ones, due to the individual layers fused together [118, 119]. Depending on the polymers used, the tablets cannot be crushed, do not disintegrate, or disintegrate very slowly, and do not exhibit abrasion [101, 120]. The porosity of the tablets can be easily controlled by the pattern and percentage of the infill of the design [120, 121]. To check the accuracy of the printing, as well as to determine the porosity of the printed tablets, µCT measurements are often used [102, 122]. The visualization of the internal structure of dosage forms reveals the structural quality, how well the layers adhere to each other, and how well the geometry matches the desired design without destruction of the tablet [123, 124]. In a study by Alhijjaj et al., it was shown that the printing speed, printing temperature, build plate leveling and polymer viscosity (melt flow index) have a high influence on the precision of the print, weight of the object and print reproducibility [125]. The effects of these parameters can be registered in the µCT and contribute to the improvement of the process.

As 3d printing is suitable for small, personalized batches, and produces a smaller throughput than industrial manufacturing machines, non-destructive methods are advantageous for this process. In addition, for the determination of the mass or content uniformity, the tablets must be dissolved, or the API must be extracted from the matrix. Therefore, there is also a growing interest in non-destructive content analysis, which is possible using NIR and Raman technology [102, 104]. This technique enables in-line and off-line implementation [126]. To verify the release of the API from the dosage form, in vitro studies must be performed. Here, the ingestion of the tablet, the residence time in the stomach and GIT are simulated. For children the dissolution studies were often adapted. For example, Starmix® candy-like dosage forms were dissolved in 2 ml saliva for 2 min, because children often are expected to chew the tablets [104, 127]. In addition, volumes and dwell times can also be adjusted for the specific patient group. Accurate dosing is especially important for children, which can be realized with FDM printing. The individually produced batch can be adapted to the needs of the children. Not only the dose, but also the release behavior can be varied. This is possible with the choice of polymer, as well as with the SA/V ratio, which can be implemented with the choice of geometry [99, 128, 129]. Various approaches are also currently being pursued to predict release curves using ANNs so that non-destructive methods can be established here as well [130–132]. These predictions are based, among other things, on the infill pattern of the tablets and their influences on the release pattern. In the study of Obeid et al. the influence of the SA/V ratio was used to predict the resulting release profile of the printed tablet [131].

## Outlook

This manuscript aims to provide an overview of pharmaceutical as well as engineering considerations for FDM printed medication for children. We reflected on current liabilities and intended to depict ways for further innovation in the engineering of unit operations to enhance suitability of equipment and dosage forms. As for 3d printing of solid dosage forms in general, formulation and print technology need to be considered in a holistic manner taking into account all aspects from raw materials to final dosage forms. We conclude that there is strong need to advance FDM printing technologies and excipients to accommodate for pharmaceutical needs—with even more elevated quality requirements for pediatric patients especially in the fields of excipient safety, acceptability, printing control and accuracy. Good news is that remedy is underway with commercial start-ups (e.g., Triastek) as well es public–private consortia such as PolyPrint actively working on the necessary technical innovation to meet pharma requirements. Also, the pediatric patient population will benefit from future capabilities of individualized therapy with precise dose adjustment and possibilities to enhance compliance via tablet morphology and size. First small clinical trials on medications for children applying other additive manufacturing techniques clearly demonstrated the future potential of the tech field [133] and we speculate that FDM—due to its technical maturity and accessibility—will be one of the key enabling technologies to advance and establish pharmaceutical 3d printing for individualized and decentralized production of dosage forms—for adults as well as children.

## Supplementary Information

Below is the link to the electronic supplementary material.

Supplementary file 1 (DOCX 43 kb)
