# Supplementary material for: Quality of FDM 3D Printed Medicines for Pediatrics: Considerations for Formulation Development, Filament Extrusion, Printing Process and Printer Design
Source: Ther Innov Regul Sci. 2021 Nov 26;56(6):910–28. doi: 10.1007/s43441-021-00354-0 (PMC9492703; doi:10.1007/s43441-021-00354-0)
Supplement: Supplementary file 1 — (DOCX 43 kb) [file 43441_2021_354_MOESM1_ESM.docx]

**Supplementary information**

Table S1: Overview of polymer requirements for the pharmaceutical application of polymers processed by HME and FDM. The authors make no claim on completeness.

| Parameter / Technique | Range / Value | Relevance  Polymer / HME /  FDM / Application |
| --- | --- | --- |
| Amorphous or semi-crystalline | Amorph: High solidification time with less shrinking | Polymer^1^ |
| API / polymer miscibility | amorphous state of API | Polymer^2^ / HME^3^ / FDM^2^ / Application^2^ |
| Brittleness  (three point bending test) | breaking distance *>* 1 mm - 1.5 mm ^3,4^  breaking stress *>* 2941 – 3126 g/mm^2 3-5^ | FDM^3,4,6^ |
| Degradation temperature *T*_deg_ | *T*_deg_ of polymer matrix has to fit thermal properties of API | Polymer^2,4,7,8^ / HME^2,4,8^ / FDM^7,8^ / Application^7^ |
| Feedability of powder | Powder in HME  Filament in FDM and for Application | HME^7^ / FDM^9,10^ / Application^9,10^ |
| Filament Diameter | Extrudate uniformity  1.75 mm | HME^11^ / FDM^11^ |
| Glass transition temperature *T*_g_ | $\frac{T_{m}}{T_{g}}$ < 1.3 preferred^8^  *T_g_* – *T*_storage_ ≥ 50 °C ^12^  50-180 °C for commonly used polymer/API combinations^2^ | Polymer^2,4,7,8^ / HME^2,4,8^ / FDM^7,8^ / Application^7^ |
| HME Temperature | Extrusion 20-30 °C below degradation temperature *T*_deg_^4^ | HME^13^ / FDM |
| HME Temperature range | 100 - 170 °C, depending on API stability | Polymer / HME^3^ |
| Low hygroscopicity | Prevents crystallization | HME^2^ / FDM^2^ / Application^2^ |
| Melt flow rate | 10 g per 10 min through heated capillary🡪 ISO 1133^3^ | HME^3^ / FDM^14^ |
| Melt viscosity *η*_melt_ | 1000 < *η*_melt_ < 10000 Pa∙s ^2,15^  viscous melt formation preextrusion^11^  solidification postextrusion^11^ | Polymer / HME^11,16^ / FDM^11,16^ / Application^11^ |
| Melting temperature *T*_m_ | $\frac{T_{m}}{T_{g}}$ < 1.3 preferred^8^  Depends on API/polymer combination  e.g. *T*_m_ (Polylactic acid) = 170 – 180 °C;^17^ *T*_m_ (Polycaprolactone) = 57 °C.^17^ | Polymer^2,4,7,8,17^ / HME^2,4,8,17^ / FDM^7,8^ / Application^7^ |
| Molecular weight |  | Polymer^2^ / HME^2^ / FDM^2^ / Application |
| No toxicity | Application of large amounts | Application^2^ |
| Particle Size |  | HME^2^ |
| Printability |  | FDM^9,10,18^ / Application^9,10,18^ |
| ratio of the elastic modulus to the viscosity of the polymer melt $\frac{\boldsymbol{\delta E}}{\boldsymbol{\eta}_{\boldsymbol{melt}}}$ | Material to be functional in FDM process with no buckling, when ratio above  $\frac{\delta E}{\eta_{melt}}$ ≥ [3 ∙ 10^5^ s^-1^ to 5 ∙ 10^5^ s^-1^] ^19^ | Polymer / HME^19,20^ / FDM^19,20^ |
| Shear thinning / thixotropy | favored | HME^3^ / FDM |
| Theoretical parameters  Flory Huggins  Hansen  PC-SAFT  COSMO | Interaction parameter (Flory-Huggins Theory):  χ <0 for strong miscibility concerning Polymer melt / API^21^  Hansen solubility parameter δ:  δ^2^ ≤ 7 MPa^½^ indicates miscibility API/polymer^21,22^  δ^2^ > 10 MPa^½^ indicates immiscibility^21,22^ | Polymer^21,22^ / HME^21,22^ / FDM^21,22^ |
| Toughness | > 80 g/mm^2^ | FDM^4^ |
| Polymer Solubility | Solvents required for processing | Polymer^23^ |
| Youngs Modulus (Tensile Test) | Youngs Modulus > 300 N/mm^2^ ^4,5^  distance at break > 1.125 mm ^5^  Thermoplastic behavior^2^ | FDM^4,5,24,25^ |

1. Penumakala PK, Santo J, Thomas A. A critical review on the fused deposition modeling of thermoplastic polymer composites. *Composites Part B: Engineering.* 2020;201.

2. K.Kolter, Karl M, Grycke A. Hot-Melt Extrusion with BASF Pharma Polymers. In: *Extrusion Compendium.* 2nd Revised and Enlarged ed.: BASF SE; 2012.

3. Cailleaux S, Sanchez-Ballester NM, Gueche YA, Bataille B, Soulairol I. Fused Deposition Modeling (FDM), the new asset for the production of tailored medicines. *J Control Release.* 2021;330:821-841.

4. Bandari S, Nyavanandi D, Dumpa N, Repka MA. Coupling hot melt extrusion and fused deposition modeling: Critical properties for successful performance. *Adv Drug Deliv Rev.* 2021;172:52-63.

5. Korte C, Quodbach J. Formulation development and process analysis of drug-loaded filaments manufactured via hot-melt extrusion for 3D-printing of medicines. *Pharm Dev Technol.* 2018;23(10):1117-1127.

6. Zhang J, Feng X, Patil H, Tiwari RV, Repka MA. Coupling 3d printing with hot-melt extrusion to produce controlled-release tablets. *Int J Pharm.* 2017;519(1-2):186-197.

7. Alshehri S, Imam SS, Hussain A, et al. Potential of solid dispersions to enhance solubility, bioavailability, and therapeutic efficacy of poorly water-soluble drugs: newer formulation techniques, current marketed scenario and patents. *Drug Deliv.* 2020;27(1):1625-1643.

8. Simoes MF, Pinto RMA, Simoes S. Hot-Melt Extrusion: a Roadmap for Product Development. *AAPS PharmSciTech.* 2021;22(5):184.

9. Nasereddin JM, Wellner N, Alhijjaj M, Belton P, Qi S. Development of a Simple Mechanical Screening Method for Predicting the Feedability of a Pharmaceutical FDM 3d printing Filament. *Pharm Res.* 2018;35(8):151.

10. Zhang J, Xu P, Vo AQ, et al. Development and evaluation of pharmaceutical 3D printability for hot melt extruded cellulose-based filaments. *J Drug Deliv Sci Technol.* 2019;52:292-302.

11. Procopio A, Tewari D. Opportunities and challenges of 3D-printed pharmaceutical dosage forms. In: *Drug Delivery Trends.*2020:15-44.

12. Agrawal AM, Dudhedia MS, Zimny E. Hot Melt Extrusion: Development of an Amorphous Solid Dispersion for an Insoluble Drug from Mini-scale to Clinical Scale. *AAPS PharmSciTech.* 2016;17(1):133-147.

13. Breitenbach J. Melt extrusion: from process to drug delivery technology. *Eur J Pharm Biopharm.* 2002;54(2):107-117.

14. Fuenmayor E, Forde M, Healy AV, et al. Material Considerations for Fused-Filament Fabrication of Solid Dosage Forms. *Pharmaceutics.* 2018;10(2).

15. Gupta SS, Solanki N, Serajuddin ATM. Investigation of Thermal and Viscoelastic Properties of Polymers Relevant to Hot Melt Extrusion, IV: Affinisol HPMC HME Polymers. *AAPS PharmSciTech.* 2016;17(1):148-157.

16. Repka MA, Bandari S, Kallakunta VR, et al. Melt extrusion with poorly soluble drugs - An integrated review. *Int J Pharm.* 2018;535(1-2):68-85.

17. Mohd Pu'ad NAS, Abdul Haq RH, Mohd Noh H, Abdullah HZ, Idris MI, Lee TC. Review on the fabrication of fused deposition modelling (FDM) composite filament for biomedical applications. *Materials Today: Proceedings.* 2020;29:228-232.

18. Korte C, Quodbach J. 3D-Printed Network Structures as Controlled-Release Drug Delivery Systems: Dose Adjustment, API Release Analysis and Prediction. *AAPS PharmSciTech.* 2018;19(8):3333-3342.

19. Venkataraman N, Rangarajan S, Matthewson MJ, et al. Feedstock material property – process relationships in fused deposition of ceramics (FDC). *Rapid Prototyping Journal.* 2000;6(4):244-253.

20. Rahim TNAT, Abdullah AM, Md Akil H. Recent Developments in Fused Deposition Modeling-Based 3d printing of Polymers and Their Composites. *Polymer Reviews.* 2019;59(4):589-624.

21. Butreddy A, Bandari S, Repka MA. Quality-by-design in hot melt extrusion based amorphous solid dispersions: An industrial perspective on product development. *Eur J Pharm Sci.* 2021;158:105655.

22. Thakkar R, Thakkar R, Pillai A, Ashour EA, Repka MA. Systematic screening of pharmaceutical polymers for hot melt extrusion processing: a comprehensive review. *Int J Pharm.* 2020;576:118989.

23. Simoes MF, Pinto RMA, Simoes S. Hot-melt extrusion in the pharmaceutical industry: toward filing a new drug application. *Drug Discov Today.* 2019;24(9):1749-1768.

24. Çevik Ü, Kam M. A Review Study on Mechanical Properties of Obtained Products by FDM Method and Metal/Polymer Composite Filament Production. *Journal of Nanomaterials.* 2020;2020:1-9.

25. Samy AA, Golbang A, Harkin-Jones E, Archer E, McIlhagger A. Prediction of part distortion in Fused Deposition Modelling (FDM) of semi-crystalline polymers via COMSOL: Effect of printing conditions. *CIRP Journal of Manufacturing Science and Technology.* 2021;33:443-453.
